# Polarization-Sensitive Light Sensors Based on a Bulk Perovskite MAPbBr_3_ Single Crystal

**DOI:** 10.3390/ma14051238

**Published:** 2021-03-05

**Authors:** Yuan Wang, Laipan Zhu, Cuifeng Du

**Affiliations:** 1 School of Civil and Resources Engineering, University of Science and Technology Beijing, Beijing 100083, China; yuanwang@ustb.edu.cn; 2 Beijing Institute of Nanoenergy and Nanosystems, Chinese Academy of Sciences, Beijing 100083, China

**Keywords:** halide perovskites, single crystal, carrier recombination, photoluminescence, light polarization

## Abstract

Organic-inorganic halide perovskites have attracted much attention thanks to their excellent optoelectronic performances. Here, a bulk CH_3_NH_3_PbBr_3_ (MAPbBr_3_) single crystal (SC) was fabricated, whose temperature and light polarization dependence was investigated by measuring photoluminescence. The presence of obvious band tail states was unveiled when the applied temperature was reduced from room temperature to 78 K. Temperature dependence of the bandgap of the MAPbBr_3_ SC was found to be abnormal compared with those of traditional semiconductors due to the presence of instabilization of out-of-phase tail states. The MAPbBr_3_ SC revealed an anisotropy light absorption for linearly polarized light with an anisotropy ratio of 1.45, and a circular dichroism ratio of up to 9% was discovered due to the spin-orbit coupling in the band tail states, exhibiting great polarization sensitivity of the MAPbBr_3_ SC for the application of light sensors. These key findings shed light on the development of potential optoelectronic and spintronic applications based on large-scaled organic-inorganic perovskite SCs.

## 1. Introduction

Organic-inorganic halide perovskites have aroused more and more interest thanks to their superior properties for potential applications in optoelectronic devices [1,2,3]. Among them, single crystal (SC) perovskites reveal better carrier transportation and stability characteristics than those from their polycrystalline counterparts, benefiting from their uniform crystal orientation, higher carrier mobility, and lower defect concentration [4]. Recently, large-sized SC perovskites grown with low-temperature solution-processed method has been proposed, which opens an avenue towards commercial applications for SC perovskites [5,6]. So far, organic-inorganic SC perovskites have found applications in solar cells [7], photodetectors [8], lasers [9], and light-emitting diodes [10], etc. Most of the former studies were basically focused on the photon-electron conversion properties of the SC perovskites.

The unveiling of much more fundamental science hidden in the SC perovskites would greatly enrich their application potentials. Recently, we have reported the influence of light-polarization on low-dimensional peroveskites such as Cs_x_(CH_3_NH_3_)_1−x_PbI_3_ nanowires and CsPbBr_3_ quantum dots [11,12]. However, the polarization dependence of the organic-inorganic perovskite single crystals is rarely reported. Polarization-sensitive light sensors for both linearly and circularly polarizations with ultra-high sensitivity based on large-scaled SC perovskites are much desired. There are still many demonstrations disclosing that low-dimensional lead halide perovskites allow a strong spin-orbit coupling (SOC), making them excellent properties in optoelectronic applications [12,13,14]. The studies for these issues in large-scaled SC perovskites are of great significance for their special applications in polarization-sensitive light sensors.

In this work, a large-scaled CH_3_NH_3_PbBr_3_ (MAPbBr_3_) SC was fabricated, and obvious band tail states were discovered under low temperature. Temperature dependence of the bandgap of the MAPbBr_3_ SC was found to be abnormal compared with traditional semiconductors. The MAPbBr_3_ SC revealed an anisotropy photoluminescence (PL) for linearly polarized light, and a circular dichroism spectrum was discovered due to the presence of spin-orbit coupling in the bands of SC. These key findings shed light on the development of potential polarization-sensitive light sensors based on large-scaled organic-inorganic perovskite SCs.

## 2. Materials and Methods

**Figure 1:**
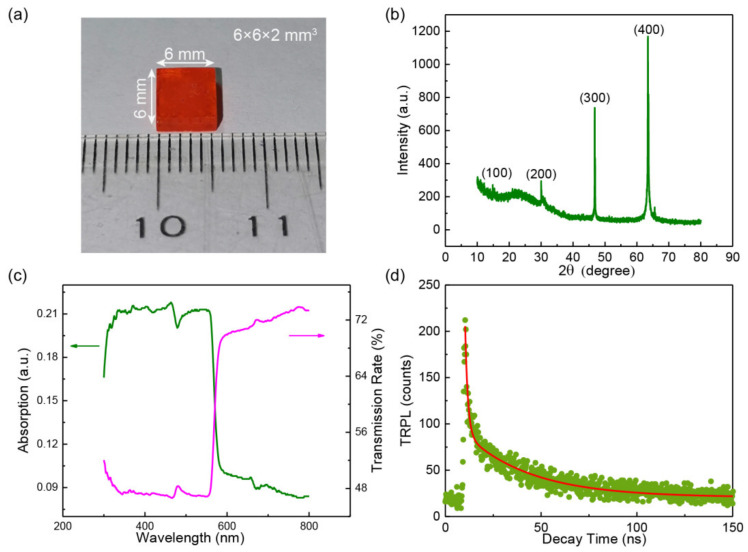
(**a**) Optical image of the as fabricated MAPbBr_3_ single crystal (SC). (**b**) X-ray diffraction (XRD) pattern of the entire MAPbBr_3_ SC. (**c**) Absorption and transmission rate spectra of the MAPbBr_3_ SC. (**d**) Time-resolved photoluminescence (TRPL) spectrum of the MAPbBr_3_ SC.

**Figure 2:**
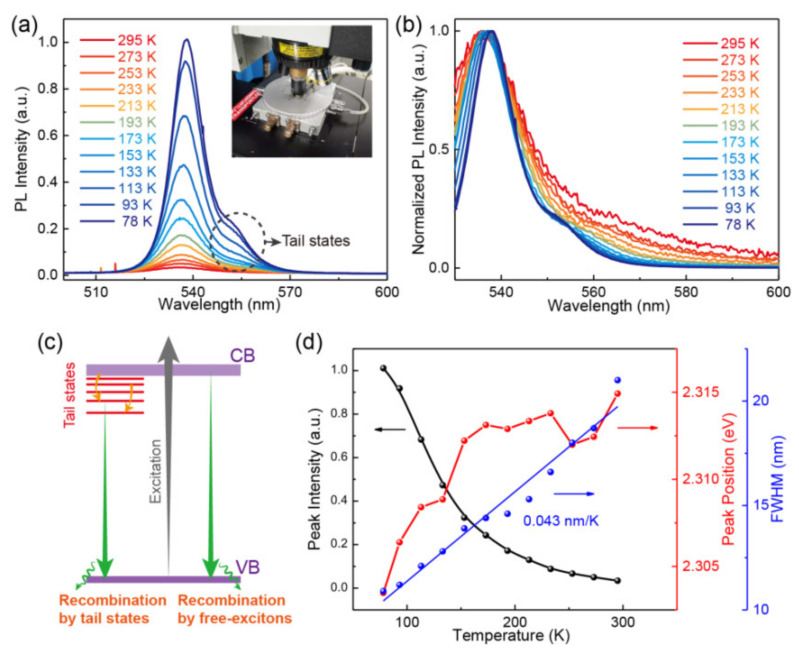
(**a**) PL spectra under different temperatures, where the inset denotes the head of the cooling equipment. (**b**) Normalized PL spectra under different temperatures. (**c**) Schematic diagram of energy band configuration showing the recombination photoluminescence mechanism. (**d**) The abstracted PL peak intensity, peak positions, and full width at half maximum (FWHM) as a function of temperature.

First, equimolar mixture of the CH_3_NH_3_Br and PbBr_2_ in 0.7 mol N,N-dimethylformamidev (DMF) were used to grow crystal seeds (with 1–2 mm in size) of MAPbBr_3_ with the above solution kept at 100 °C for 24 h, where the CH_3_NH_3_Br, PbBr_2_ and DMF were purchased from Nanjing MKNANO Tech. Co., Ltd., Nanjing, Jiangsu, China. By putting one of the seeds into a fresh solution kept still at 100 °C for 48 h, a large-sized crystal of ~6 × 6 × 2 mm^3^ was then fabricated, as shown in Figure 1a, which displayed an orange color under natural light. The single crystal was stored in a vacuum drying vessel to protect it from being decomposed before further investigation. The X-ray diffraction (XRD) pattern obtained with a PANalytical X’Pert^3^ Powder X-ray diffractometer (Malvern Panalytical, Westborough, MA, USA) shows obvious crystal faces corresponding to (100), (200), (300), and (400) indices (see Figure 1b), indicating a good single crystal quality of the MAPbBr_3_ with [100] crystal orientation pointing away from the upper surface. The weaker (100) peak compared with the work of Liu et al. is due to that the measured sample is an entirely bulk crystal. but not pretreated powder [5]. Compared to those with higher crystal indices, the relatively poor morphology of the (100) plane results in a weak peak. The absorption and transmission rate spectra were measured using a UV3600 UV-vis-NIR spectrophotometer (Shimadzu, Kyoto, Japan). The changing tendencies of the absorption and transmission rate spectra were basically opposite, and the absorption onset of 574 nm was observed (Figure 1c), demonstrating a large absorption coefficient and a low transmission rate when the wavelength was below 574 nm due to the intrinsic energy band structure of the SC MAPbBr_3_. A quick recombination of 27 ns on average was acquired via the measurement of time-resolved photoluminescence (TRPL) spectrum using a FLS980-S2S2-stm spectrometer (Edinburg Instruments Ltd., Edinburgh, UK) with the excitation source of 375 nm pulse laser (see Figure 1d), indicating the photoluminescence of the SC MAPbBr_3_ was mainly resulted from exciton recombination [15,16]. The PL spectra were measured using a confocal Raman/PL spectroscope (HORIBA/LabRAM HR Evolution, Paris, France), and a cooling process ranging from 295 to 78 K was controlled by a Linkam’s HFS probe system (HFS600E-PB2, Tadworth, UK), as shown in Figure 2a. The excitation light for the temperature dependent PL spectra was a 325 nm continuous wave laser with a constant output power of 1.5 mW and a diameter of 2 mm.

## 3. Results and Discussion

From Figure 2a, we can see the PL intensity is increased with the decrease of temperature. The PL spectra show more and more apparent right shoulders around their peaks with the decrease of temperature, which is believed to derive from the concurrence of free-exciton states and bright tail states that causes diverse recombination luminescence probability of excitons localized in the states [17,18]. The enhanced right shoulder in the PL spectra results from the accelerated recombination by the bright tail states below the conduction band (CB) of the MAPbBr_3_ SC. In order to display the tail states much more clearly, normalized PL spectra are presented in Figure 2b. One can see the tail states start appearing at 153 K, which corresponds to the temperature of structural phase-changing from a tetragonal structure to an orthogonal structure [19]. It reveals that the tail states might be derived from the specific electronic structure of the orthogonal structure. A schematic energy band is depicted to explain the interesting shape under lower temperatures (see Figure 2c). When a light with energy surpassing the band gap irradiates on the surface of MAPbBr_3_ SC, there will be electron-hole pairs excited in the conduction and valence bands, respectively. Then, the exited free electrons and holes will relax to the bottom of the two bands and mainly get recombination through the form of free-excitons at room temperature. With the decrease of temperature, the instabilization of out-of-phase tail states will be present, hence more and more electrons will relax to the tail states, and as a result, the recombination due to the tail states becomes much more significant [12,17]. The full width at half maximum (FWHM) of the PL spectra is around 20 nm at room temperature and decreases linearly with a slope of 0.043 nm/K when the temperature further reducing (see Figure 2d), which reveals that the MAPbBr_3_ SC can produce much more saturated colors compared with traditional light emitting diodes, and hence possesses better promising applications in displays as well as temperature sensors [20,21,22]. The temperature dependence of the PL intensity and that of the peak position are abstracted in the black and red labels in Figure 2d, respectively. The recombination luminescence exhibits a redshift when decreasing temperature from 295 to 78 K, which is reversed to those observed from traditional semiconductors such as Si, Ge, and GaAs [23]. The bandgap of MAPbBr_3_ SC can be expressed by

*E*_g_ = *E*_0_ + *α*_g_*T*, (1)

where *E*_0_ is the bandgap corresponding to 0 K, *T* is the applied temperature, and *α*_g_ is a positive temperature coefficient [24]. The two different slopes below and above 170 K might be due to different phase structures. The peak positions obtained here mean the PL energies corresponding to the maximum intensity. For higher temperatures above 233 K, the peaks are much wider and their intensity is much weaker, and as a result, the maximal values abstracted might be larger deviations with the actual peak positions. Hence a non-continuous redshift is observed.

**Figure 3:**
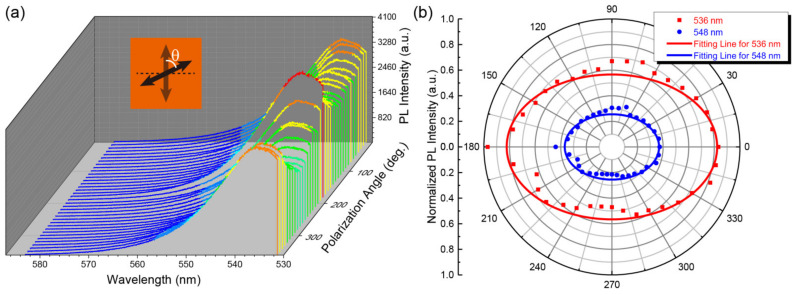
(**a**) Waterfall plot of PL spectra with different incidence polarization angles at room temperature. (**b**) Polarization angle dependence of the normalized PL intensity for luminescence wavelength of 536 and 548 nm, respectively.

Then, we studied the PL spectra as a function of linear polarization angle of the excitation light irradiating vertically on the surface of the single crystal at room temperature, as shown in Figure 3a. Noting that, as it is not feasible to tune the polarization states of the 325 nm laser light in our present equipment, the excitation light had to be changed to a 532 nm laser, whose polarization states could be easily manipulated through a half wavelength plate in the optical path system, and the 532 nm laser spot was focused to be ~2 mm in diameter. Although the excitation sources are different, both of them correspond to the interband excitation transitions of the single crystal, hence the essential physical phenomena obtained could be compared. From Figure 3a, we can see that the PL intensity shifted with different linear polarization angles. The normalized PL intensity at different polarization angles corresponding to 536 and 548 nm were abstracted and fitted with a normal elliptical formula (Figure 3b). There is an anisotropic PL within (00n) crystalline plane (where *n* = 1, 2, 3, …) with the anisotropy ratio of 1.45:

(2) $$A=\frac{P_{max}}{P_{min}}$$

where *P*_max_ and *P*_min_ are the maximum and minimum PL intensity, respectively. This PL anisotropy is deemed to derive from the anisotropy of light absorption [25,26,27]. The deviation between the raw data and the fitting curve is probably due to the surface defects of the single crystal. However, at least, it unveils that the MAPbBr_3_ SC possesses application potentials in linear polarization detectors after an optimized fabrication of crystal surface.

**Figure 4:**
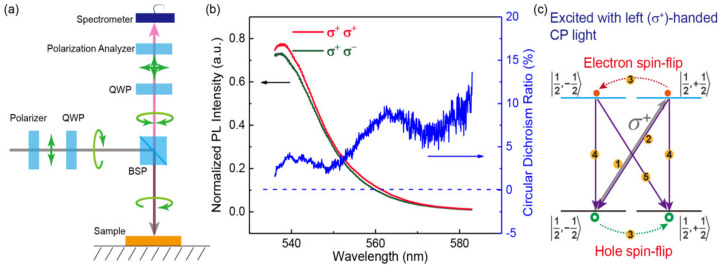
(**a**) Schematic optical path configuration for the measurement of circular dichroism. (**b**) The normalized circularly polarized PL intensity and the circular dichroism ratio as a function of luminescence wavelength when a left-handed polarized light is applied as an excitation light. (**c**) Proposed mechanism for the circular dichroism PL.

Next, circular polarization dependence of the PL spectra was studied, the optical system is schematically shown in Figure 4a. A linearly polarized light was aligned after the incidence light going through a polarizer, and a circularly polarized light was produced when the linearly polarized light went through a quarter wave plate (QWP), which was reflected to the surface of the single crystal with a beam splitter (BSP), then the left (*σ*^+^) and right-handed (*σ*^−^) circularly polarized lights went through the BSP and a QWP with the same angle of fast axis as the one in the incidence light path. The left- and right-handed circularly polarized lights became linearly polarized lights with polarization directions perpendicular to each other whose intensity were detected by a spectrometer after the PL light going through a polarization analyzer (see Figure 4a). Noting that the PL spectra corresponding to the two circular polarization states could not be analyzed simultaneously, they had to be measured manually and respectively by rotating the angle of the polarization analyzer. When a left-handed polarized light was used to irradiate on the SC surface, the PL spectra intensity corresponding to left- and right-handed lights were different, as shown in Figure 4b. In other words, there is a circular dichroism of PL. A circular dichroism ratio is determined by

(3) $$P=\frac{I_{\sigma^{+}\sigma^{+}}-I_{\sigma^{+}\sigma^{-}}}{I_{\sigma^{+}\sigma^{+}}+I_{\sigma^{+}\sigma^{-}}},$$

where $I_{\sigma^{+}\sigma^{+}}$ and $I_{\sigma^{+}\sigma^{-}}$ are the PL intensity of *σ*^+^ and *σ*^−^ circularly polarized lights with *σ*^+^ light incidence [13,28]. The wavelength dependence of the circular dichroism ratio in Figure 4b reveals that the circular dichroism ratio is increased basically with the wavelength increasing and appears maximal at values of ~540 and ~565 nm, which might be a result from the spin-orbit coupled recombination luminescence corresponding to the inner band and tail states, respectively, and the circular dichroism ratio can reach up to 9% for recombination luminescence due to the tail states at 565 nm. What is more important, although the tail states are indistinguishable at room temperature in a single PL spectrum for both linearly and circularly polarized light excitations, the investigation of circular dichroism can make it possible to detect the tail states.

A schematic diagram is proposed in Figure 4c to illustrate the possible mechanism for the spin-orbit coupled recombination luminescence. Benefiting from the existence of heavy elements (Pb, Br), a large Rashba SOC will be induced, i.e., electron spins entangled with the p-orbitals of the Pb and meanwhile hole spins entangled with the s-orbital of the Pb and p-orbitals of the surrounding Br [29]. A spin-splitted conduction band (CB) and a spin-splitted valence band (VB) with the same spin states of $|\frac{1}{2} ,-\frac{1}{2}\rangle$ and $\left|\frac{1}{2}\right.,+\frac{1}{2}\rangle$ will be evoked due to the strong Rashba SOC [12,29,30]. When a left-handed circularly polarized light is irradiated on the surface of the single crystal, it will excite spin-polarized electrons with spin-up state of $\left|\frac{1}{2}\right.,+\frac{1}{2}\rangle$ in the CB and spin-polarized holes with spin-down state of $\left|\frac{1}{2}\right.,-\frac{1}{2}\rangle$ in the VB, respectively (process 1 in Figure 4c). The spin-polarized electrons will get recombined back from CB $\left|\frac{1}{2}\right.,+\frac{1}{2}\rangle$ to VB $\left|\frac{1}{2}\right.,-\frac{1}{2}\rangle$, resulting in a left-handed recombination luminescence (process 2 in Figure 4c). At the same time, the spin-up electrons and spin-down holes will also experience spin flippings due to spin-related scatterings, which correspond to process 3 in Figure 4c. The spin-flipped carriers will get recombined from CB $\left|\frac{1}{2}\right.,-\frac{1}{2}\rangle$ to VB $\left|\frac{1}{2}\right.,+\frac{1}{2}\rangle$, as a result a right-handed recombination luminescence (process 5 in Figure 4c) will happen. As the number of excitons recombined with processes 2 and 5, respectively, are different, a circular dichroism is found (Figure 4b). Process 4 in Figure 4c represents the recombination corresponding to intersubbands with the same spin angular momentum, which will lead to a luminescence of linearly polarized light. In general, there are a large proportion of linearly polarized light in the normalized PL spectra of Figure 4b, which is the reason why the two spectra are basically uniform in amplitude. However, the amplitude difference of the two spectra is enough for demonstrating the presence of strong spin-orbit couplings corresponding to both the intersubbands and the tail state bands, and a larger circular dichroism ratio for the tail state bands indicates that the Rashba SOC splitting in the tail state bands might be much larger than that in the intersubbands. For the case of excitation with a right-handed CP light, it is similar with that of the left-handed one, which will firstly excite electrons and holes in CB $\left|\frac{1}{2}\right.,-\frac{1}{2}\rangle$ and VB $\left|\frac{1}{2}\right.,+\frac{1}{2}\rangle$, respectively. As the intensity of right-handed luminescence is stronger than that of right-handed luminescence, a circular dichroism is expected to be found.

## 4. Conclusions

In conclusion, a large-scaled organic-inorganic halide bulk MAPbBr_3_ single crystal was fabricated, and the temperature and light polarization dependence of the single crystal was studied in detail. With the decrease of temperature, the PL intensity was increased, and the PL peak positions revealed a redshift due to the instabilization of out-of-phase tail states. The FWHM of the PL spectra is ~20 nm at room temperature and decreases linearly with a slope of 0.043 nm/K when the temperature is further reduced. The MAPbBr_3_ SC reveals an anisotropy ratio of 1.45 for linearly polarized light excitation due to anisotropy light absorption. A circular dichroism spectrum is discovered due to the spin-orbit coupled recombination luminescence, which can be used for a judgment for the presence of tail states at room temperature, and the circular dichroism ratio can reach up to 9% for recombination luminescence due to the presence of tail states. These key findings shed light on the development of potential polarization light sensors and spintronic devices based on large-scaled perovskite single crystals.

## Data Availability

Data is contained within the article.
